# Maltodextrin-modified lipoplexes for enhanced mucosal penetration and efficient mRNA delivery

**DOI:** 10.1016/j.mtbio.2025.101975

**Published:** 2025-06-13

**Authors:** Bojan Kopilovic, Nabila Laroui, Mathieu Berchel, Paul-Alain Jaffrès, Patrick Midoux, Mara G. Freire, Chantal Pichon

**Affiliations:** a CICECO - Aveiro Institute of Materials, Department of Chemistry, University of Aveiro, 3810-193, Aveiro, Portugal; b Centre de Biophysique Moléculaire (CBM), UPR 4301 CNRS, F-45071, Orléans, France; c Inserm US 55 ART ARNm and University of Orléans, F-45100, Orléans, France; d Institut Universitaire de France, 1 rue Descartes, F-75035, Paris, France; e Univ Brest, CEMCA, UMR CNRS - UBO 6521, F-29200, Brest, France

**Keywords:** Imidazolium-based lipids, Maltodextrin, Nasal delivery, Mucosal penetration, Lipoplexes

## Abstract

Efficient delivery of messenger ribonucleic acid (mRNA) to mucosal tissues represents a promising approach for localized protein production in the nasal and respiratory tract. Here, we investigate the use of maltodextrin (MDX) as a surface modifier to enhance the delivery of mRNA-loaded histidylated lipoplexes (LXs) to airway epithelial cells. By reducing hydrophobicity, MDX facilitates better penetration through the mucus layer, enabling effective mRNA delivery. MDX-coated LXs improve mRNA delivery and expression *in vitro* by increasing cellular uptake and supporting sustained protein production. Additionally, MDX incorporation stabilizes in-house-formulated lipoplexes and modulates their interactions with mucin-covered cells. Notably, MDX-coated mRNA LXs display a four-fold increased transfection efficiency, and the protein expression is maintained up to 48 h post-transfection. Furthermore, intranasal administration of MDX-LXs results in efficient gene expression *in vivo*. Overall, our findings reveal that integrating MDX into mRNA lipoplexes is a promising strategy to advance nasal delivery for gene therapy and protein replacement applications.

## Graphical abstract

**Figure:**
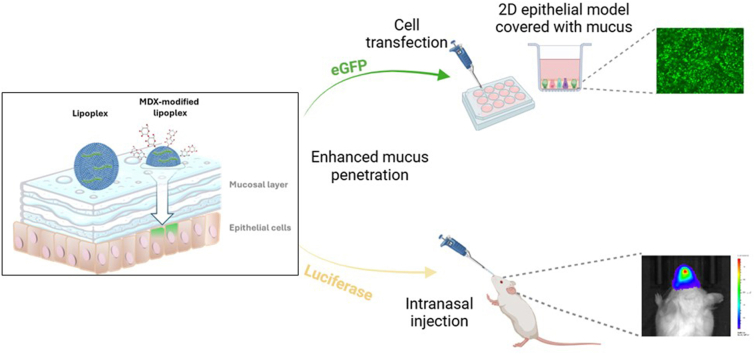

## 1 Introduction

Recent advances in nanotechnology have enabled the development of efficient platforms for delivering nucleic acids such as messenger RNA. *In vitro* transcribed messenger ribonucleic acid (IVT-mRNA) holds immense potential to address clinical needs that have not yet been met with existing solutions [1]. IVT-mRNA provides a flexible and potent tool for inducing transient protein expression in targeted tissues. A key challenge in translating this potential into clinical applications lies in designing effective delivery systems capable of protecting mRNA and facilitating its uptake and translation in target cells [1].

Delivering mRNA to the respiratory tract poses unique challenges due to the complex architecture and protective mucus barrier of the airway epithelium. The intricate structure of the respiratory tract and its hostile microenvironment, which is designed to eliminate foreign particles, further hinder efficient mRNA delivery. In addition, the shear force from nebulization and the heterogeneous nature of airway cells pose further challenges [2].

Traditional delivery systems may face limited penetration, instability, and inefficient cellular uptake in this environment. To overcome these obstacles, researchers have turned to specialized carriers and surface modifications aimed at enhancing mucosal permeability and intracellular delivery efficiency. A variety of polymers and biomaterials have been utilized for mucosal mRNA delivery, using their tailored physical and chemical properties to enhance transport across the mucus layer [3,4]. Polymeric modifications are a common strategy that can be used to adjust charge, hydrophobicity, and the ability to adhere to mucus, resulting in increased retention and diffusion of antigen-carrying carriers through the mucosa. Polyethylenimine (PEI) has been demonstrated to function as a powerful mucosal adjuvant for intranasal administration [5,6]. Nevertheless, challenges in maximizing its adjuvant effect while minimizing its toxicity persist [6]. Optimizing its chemical structure appears to be a practical solution. The chemical conjugation of cyclodextrin with PEI, as reported by Li et al. [5], was shown to enhance both humoral and cellular immune responses. Additionally, the same cyclodextrin–PEI formulation was evaluated for its ability to penetrate the airway epithelial barrier and deliver mRNA encoding HIV gp120 paracellularly [7]. The results showed potent systemic and mucosal anti-HIV immune responses following intranasal administration, likely due to prolonged nasal cavity residence time and excellent intracellular delivery. The presence of cyclodextrin in the conjugate allows greater mucosal adhesion than PEI alone, leading to longer nasal retention time of the nanoparticles (NPs), which further increases uptake by nasal-associated lymphoid tissue and nasal epithelial cells [8].

In this work, we produced different liposomes to complex mRNA encoding enhanced green fluorescent protein (eGFP) and added maltodextrin (MDX) to foster their delivery in airway epithelial cells in the presence of mucin. MDX is an innocuous oligosaccharide to human health, of low cost and readily soluble at physiological pH, making it a versatile ingredient for many applications, including the development of healthcare products. Our rationale is to have a straightforward mixing or incubation process that requires no chemical reactions. This avoids complex synthesis protocols and eliminates the need for purification of conjugates. Moreover, non-covalent coatings preserve the original structure and function of both the delivery system and the modifying agent.

We used several liposome formulations prepared by lipid combinations (KLN25, MM27, DOPE, cholesterol, β-sitosterol, DMPE-PEG and DMG-PEG) for mRNA delivery. This plethora of lipids offer specific properties that contribute to an efficient delivery of mRNA [9]. Cationic lipid KLN25 (*O*,*O*-dioleyl-*N*-[3-*N*-(*N*-methylimidazolium iodide)propylene] phosphoramidate) facilitates mRNA complexation and protection against RNase [10]. Ionizable lipids such as MM27 (*O*,*O*-dioleyl-*N*-histamine phosphoramidate) have a histidine polar head and an imidazole group that become protonated at pH < 6.0. This effect promotes the fusogenic properties of liposomes with the endosomal membrane causing destabilization and the cytosolic delivery of mRNA [10]. DOPE acts as a neutral lipid, promoting the disruption of the endosomal membrane, improving mRNA intracellular delivery and its translation [11]. Cholesterol, on the other hand, is commonly used for stabilizing lipid bilayers; however, it lacks fusogenic properties [11]. Alternatively, β-sitosterol could be used as well and it is reported to not alter particle size nor encapsulation efficiency, yet it holds the potential to improve translation efficiency in some conditions [11]. Ultimately, PEGylated lipids are used to prevent aggregation of nanoparticles [10]. The importance of choosing the right combination of lipids lies in their roles in mRNA complexation, protection, and membrane destabilization, ultimately enhancing transfection efficiency in various cell lines. We finally evaluated the impact of MDX on the physicochemical features of lipoplexes, uptake by human airway epithelial cells, intracellular localization, and transfection efficiency. It is shown that the addition of MDX is highly beneficial in terms of size stabilization throughout one month of storage, gene expression kinetics and intracellular mRNA trafficking in general.

## 2 Materials and methods

### 2.1 Reagents

Maltodextrin (MDX) (dextrose equivalent 4.0–7.0) was purchased from Sigma Aldrich. *O,O*-dioleyl-*N*-[3*N*-(*N*-methylimidazolium iodide)propylene] phosphoramidate (KLN25; MW = 787 Da) and *O,O*-dioleyl-*N*-histamine phosphoramidate (MM27; MW = 692 Da) were synthesized as described elsewhere [12]. 1,2-dimyristoyl-*sn*-glycero-3-phosphoethanolamine-*N*-[methoxy(polyethylene glycol)-2000] (DMPE-PEG; MW = 2693.32 Da). Cholesterol (MW = 386.65 Da), β-sitosterol (MW = 414.71 Da), DMG-PEG 2000 (1,2-dimyristoyl-*sn*-glycero-3-phosphoethanolamine; MW = 2509.20 Da), and 1,2-di-(9Z-octadecenoyl)-*sn*-glycero-3-phosphoethanolamine (DOPE; MW = 744.034 Da) were purchased from Avanti Polar Lipids (Avanti, USA). The chemical structure of the used lipids is shown in Figure S1. Lipofectamine Messenger Max™ (LFM) was purchased from Invitrogen (Thermofisher, France). Lyophilized mucin from bovine submaxillary glands was purchased from Sigma-Aldrich, France. Modified mRNA (with 5-methoxyuridine and containing CleanCap) encoding the enhanced green fluorescence protein (eGFP) was purchased from Tebu-bio (France). Modified mRNA (with 5-methoxyuridine and containing Cap-1 encoding Firefly Luciferase (F-Luc) was purchased from Oz Biosciences (France).

### 2.2 *In vitro* transcription of eGFP-mRNA

Anti-reverse cap analog (ARCA)-capped RNA with a poly(A) tail coding the reporter gene eGFP was produced by *in vitro* transcription using the T7 mMessage mMachine Ultra kit as described in Mockey et al. [13] The RNA concentration was determined by absorbance at 260 nm; RNA had 260:280 ratio ≥2 and was stored at −80 °C in small aliquots. The quality of eGFP mRNAs was verified by capillary electrophoresis using Agilent 5400 Fragment analyzer.

### 2.3 Fluorescence labeling

mRNA was labeled either with FITC or Cy5 probe using Label IT® Nucleic Acid Labeling Reagents (Mirus Bio LLC, Madison, WI, USA), following the manufacturer's instructions. Rhodamine-labeled liposomes were produced by incorporation of rhodamine-labeled-DOPE (Rho; Avanti Polar Lipid). Fluorescein-labeled maltodextrin (FITC-MDX) was obtained as previously reported by De Belder et al. [14] Briefly, 10 g of MDX were dispersed in 100 mL of anhydrous DMSO, which contained a few drops of pyridine, and then 25 mg of fluorescein-isothiocyanate (FITC) was added, followed by 200 mg of dibutyltin di-laureate. The mixture was stirred gently at 65 °C for 2 h. FITC-MDX was then precipitated with 95 % v/v ethanol, and the precipitate was washed several times with ethanol to remove any residual free dye. Finally, the FITC-MDX was freeze-dried, and the powder was stored at 4 °C in containers protected from light.

### 2.4 Liposomes preparation

Liposomes were prepared using the lipids described in Table 1. For lip1, 0.5 mL of a 5.4 mM KLN25 solution in ethanol was mixed with 0.5 mL of a 5.4 mM MM27 solution in ethanol. The ethanol solutions were then evaporated at 60 °C (0 bar) in a rotary evaporator (for 30 min) until a film was formed. The film was hydrated in 0.5 mL of 10 mM Hepes buffer (pH 7.4) for 12 h at 4 °C, followed by vortexing and sonication for 15 min at 37 kHz using a Bioblock ultrasonic bath (Bioblock Scientific, Illkirch, France). The resulting liposomes (500 μL) were dialyzed against 500 mL of HEPES at 4 °C for 6 h using dialysis tubing with a cellulose membrane (MWCO: 12.4 kDa), this stems from an in-house protocol. Lip2 and lip3 liposomes were prepared using their respective lipids dissolved in ethanol to achieve a known concentration of mM solution (21.6 and 5.4, respectively, for lip2 and lip3). Liposomes were prepared using Dulbecco's phosphate-buffered saline (DPBS) (Thermo Fisher, France), with a volume ratio of 1:3 between the ethanol phase and the aqueous phase. The two phases were mixed rapidly at a total flow rate of 10 mL/min using a NanoAssemblr® Ignite™ microfluidic chip (NxGen microfluidics, Precision NanoSystems, Canada). The resulting products were dialyzed against DPBS at 4 °C for 6 h using dialysis tubing with a cellulose membrane (MWCO: 10 kDa). To prepare fluorescent liposomes, 0.5 % (molar ratio) of rhodamine-lipid was added to the lipid mixture prior to microfluidic mixing.

**Table 1:** Liposome formulations (mole ratio).

	KLN25	MM27	Cholesterol	DMPE-PEG	DMG-PEG	β-sitosterol	DOPE
**lip1**	50	50	–	–	–	–	–
**lip2**	20	40	38.5	1.5	–	–	–
**lip3**	20	40	–	–	1	29	10

### 2.5 Lipoplex preparation and characterization

#### 2.5.1 mRNA complexation

eGFP mRNA-LX were prepared by mixing eGFP mRNA and liposomes at various mRNA/liposome (RNA/L) charge ratios (1/0.5, 1/1, 1/1.5, 1/2) and leaving them for 15 min at room temperature. Using the same procedure, LX-MDX were prepared by mixing eGFP mRNA and MDX (at a concentration of 1 mg/mL in HEPES) at RNA/MDX weight ratio of 1:2, and then the apropriate amount of liposomes was added at desired mRNA/L charge ratio. Several RNA/MDX weight ratios have also been screened, namely 1/0.5, 1/1 and 1/2.

#### 2.5.2 Encapsulation efficiency

The Ribogreen assay was used to analyze the mRNA encapsulation efficiency in liposomes. Briefly, lipoplex samples and mRNA concentration standards were produced in Tris-EDTA (TE) buffer with and without Triton X-100. These were placed in a black 96-well plate, followed by the addition of Ribogreen dye, which fluoresces when bound to RNA. Fluorescence was measured with a plate reader (ClarioStar BMG), (λ_ex_ = 485 nm, λ_em_ = 528 nm). The percentage of mRNA encapsulation was estimated as the ratio of the average fluorescence intensity in TE buffer to the average fluorescence intensity in TE buffer containing Triton X100, in triplicate.

#### 2.5.3 Electrophoretic gel retardation assay

mRNA complexation with liposomes was monitored by electrophoresis to assess LX formation at different ratios through a 0.6 % agarose gel containing 0.02 % of Ribogreen (ThermoFisher, France). Each well was loaded with samples comprising 100 ng of eGFP mRNA. Gels were imaged using a GelDocXR + Imager (Biorad, Hercules, CA, USA).

#### 2.5.4 Size and zeta potential

Hydrodynamic diameter and polydispersity index (PDI) of formulations were measured in DPBS by Quasi-elastic laser light scattering with the SZ100 NanoPartica (Horiba Scientific, France). Measurements were calibrated with 204 nm latex nanosphere size standards and DTS 1050 standard. The zeta Potential of formulations was measured in NaCl buffer at 10 mM by electrophoretic mobility using zetaSizer Ultra Red (Malvern, France). Each sample was prepared with 5 μg eGFP mRNA and diluted 250 times.

### 2.6 Preparation of mucin samples

To prepare a 0.5 % w/v mucin solution, 50 mg of lyophilized mucin from bovine submaxillary glands was dissolved in 10 mL of DPBS. The mixture was vortexed at 25 °C until the mucin was completely dissolved. The airway mucin hydrogel was created by dissolving mucins in DPBS buffer at a concentration of 2.5 % (w/v) at 25 °C. The solution was stirred for 1 h before being used [3].

### 2.7 Cell culture

NCI-H292 airway epithelial cell line was a gift from Professor Didier Betbeder (Vaxinano, Lille, France). The cells were cultured in RPMI 1640 medium (Thermo Fisher Scientific, France) supplemented with 10 % heat-inactivated fetal bovine serum (FBS), 100 U/mL penicillin, 100 μg/mL streptomycin, and 1 % (v/v) L-glutamine (Thermo Fisher Scientific, France) at 37 °C in a 5 % CO_2_ humidified atmosphere. Cells were mycoplasma-free as evidenced by the MycoAlert Mycoplasma Detection Kit (Lonza, Levallois Perret, France).

### 2.8 mRNA transfection

Cells were seeded at a density of 1.2 × 10^5^ cells *per* well in 24-well plates 24 h prior to the treatment. This density is higher than commonly reported in the literature and thus required an increased amount of mRNA to achieve effective transfection across the entire monolayer cell population. In contrast to previous studies [3], the cells were incubated with mucin to naturally coat them, prior to assessing their impact on LX uptake. For that, cells were previously incubated with 500 μL mucin (LX:mucin ratio of 1:5 (v/v)) of 0.5 mg/mL mucin in of fresh medium for 10 min at 37 °C and subsequently exposed to the LX containing eGFP-mRNA. Medium was replaced after 4 h with a fresh one containing 10 % FBS. Transfection efficiency was evaluated after 24 and 48 h. Cells were harvested by using trypsin (Thermo Fisher Scientific, France), collected by centrifugation, and diluted in DPBS. Just before fluorescence measurement, cells were gently mixed and propidium iodide (PI) was added at a final concentration of 0.01 mg/mL The percentage of transfected cells (%), the mean of the fluorescence intensity (MFI) and the mortality (PI positive cells) were measured with a flow cytometer (FORTESSA X20; Becton Dickinson, Franklin Lakes, NJ, USA) with λ_ex_ = 488 nm; λ_em_ = 530 nm for fluorescein and λ_ex_ = 537 nm; λ_em_ = 618 nm for PI. The fluorescence intensity was expressed as the mean fluorescence intensity of 10,000 events.

### 2.9 Cellular uptake assay

The uptake of formulations was performed as described by Delehedde *et a*l. [15]. Briefly, 1.2 × 10^5^ NCI-H292 cells were plated in a 24-well plate the day before the experiments. LXs were prepared with FITC-labeled mRNA. Cells were then incubated with LX for varying duration times in serum-free medium. Subsequently, the medium was discarded by aspiration, and cells were rinsed with cold PBS before detachment in 600 μL PBS. The cell suspension was transferred to round-bottom polystyrene tubes and maintained on ice. Fluorescein fluorescence intensity was measured by flow cytometry with FORTESSA X20 (Becton Dickinson, Franklin Lakes, NJ, USA) and expressed as the mean value of the fluorescence intensity (MFI) of 10,000 events.

To determine LX binding on the cell surface, the MFI associated with each sample was measured both before and after treatment with trypan blue (TB) at a final concentration of 0.004 % to quench the fluorescence of fluorescein-labeled NPs bound on the cell surface. The difference between MFI before and after TB treatment represented the amount of NPs bound on the cell surface (surface). To determine the total uptake of NPs and their localization within acidic compartments, the cells were treated with monensin (0.0354 mg/mL in PBS, pH 7.4) for 30 min on ice. Monensin, acting as an ionophore, permeabilizes cells, neutralizing intracellular acidic compartments. MFI enhancement upon monensin treatment reflects the amount of NPs residing in acidic environments. The total uptake is the difference between MFI after monensin treatment and MFI from the surface binding.

### 2.10 Confocal microscopy analysis

The day before experiments, 8.5 × 10^3^ cells were seeded in an 8-well Lab-Tek chambered cover glass (Nunc, Dutsher S.A., Brumath, France). Cells were washed three times with serum-free medium and stained with 20 μM Hoechst 33258 dye (Eurogentec, Belgium) for 10 min to label nuclei. Subsequently, LXs made with Cy5-labeled mRNA, rhodamine-labeled liposomes, and FITC-labeled MDX were added to the cells, in media containing mucin. The cells were then incubated at 37 °C for specific time intervals (15, 20, 40, 60, and 120 min) and subsequently visualized using a Zeiss Axiovert 200 M microscope coupled with a Zeiss LSM 980 airyscan 2 (Carl Zeiss Co., Ltd., Iena, Germany). The inverted microscope is equipped with a temperature-controlled stage and a Plan-Apochromat 63 × objective (NA ¼ 1.4). Images were analyzed with Zeiss software ZEN.

### 2.11 Cryo-electron microscopy analysis

For cryo-EM imaging, 3.5 μL of samples were deposited on C-Flat holey carbon grids (CF-2/2-3Cu-T-50, Protochips, Morrisville, NC, USA) and vitrified with a Vitrobot Mark IV set at 4 °C and 100 % humidity (Thermo Fischer, Waltham, MA, USA). Grids were then transferred onto a cryo holder (Cryo-Holder Fischione model 2550, Fischione Instruments, Export, PA, USA) before observations under a transmission electron microscope (JEOL 1400 Plus, Tokyo, Japan). Cryo-EM images were collected at 120 kV at a temperature of 100 K and taken at 1.5–2 μm defocus to improve contrast. The used camera was OneView (Gatan, Pleasanton, CA, USA).

### 2.12 *In vivo* assays

Specific pathogen-free female BALB/c (7–8 weeks) mice were obtained from Charles River (France) and kept in isolated ventilated cages. All animal studies were approved by the French Ministry of Agriculture for experiments with laboratory animals (#31284). Mice received an intranasal injection of 30 μL of LX3 and LX3-MDX comprising 10 μg F-Luc mRNA and were imaged 6, 24 and 48 h post injections. On the imaging day, mice received an intraperitoneal injection of 100 μL of D-luciferin Bioluminescent Substrate (0.15 g/kg body weight) (Promega, Madison, WI, USA). Following the injection, mice were allowed a 7-min rest period before being subjected to anesthesia (2.5% isoflurane) for a duration of 3 min. Approximately 10 min after the administration of the D-luciferin substrate, mice were subjected to imaging using the IVIS Lumina system Imaging system (Revvity, Bussy-saint-martin, France). Post-imaging, the acquired signal was quantified utilizing Living Image® 4.5.5 software (IVIS Imaging Systems) and expressed as total flux (photons per second).

### 2.13 Statistical analysis

Statistical analysis was performed in Excel stat software. All data are presented as mean ± SD. Data of two groups were compared using a two-tailed Student's t-test. P values less than 0.05 were considered statistically significant (∗p < 0.05, ∗∗p < 0.01, ∗∗∗p < 0.001).

## 3 Results and discussion

### 3.1 Lipoplex preparation and characterization

To ensure effective delivery of mRNA, it is essential for the delivery system to protect it from premature degradation. The interaction between negatively charged phosphate groups of mRNA and positively charged amine groups of lipids allows the formation of complexes designated as lipoplexes (LXs), enabling the mRNA to be protected from degradation by RNases [16]. In this study, three different liposomes were produced, as summarized in Table 1. All of them contain the same cationic lipid (KLN25) and ionizable lipid (MM27), but at different mole ratios. lip1 contains only KLN25 and MM27 (50:50) whilst lip2 and lip3 are comprised of KLN25 and MM27 at a ratio of 20:40. They also differ by the presence of DMPE-PEG and cholesterol for lip2, while lip3 comprises DMG-PEG, β-sitosterol and DOPE.

**Fig. 1:**
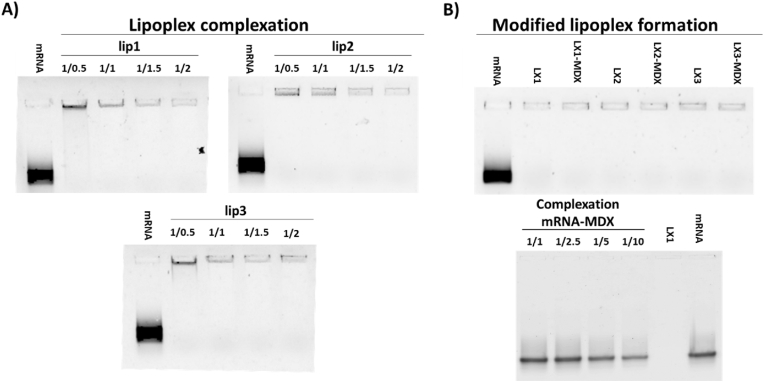
Evaluation of mRNA complexation by agarose gel electrophoresis. (**A**), mRNA complexation: free mRNA and lipoplex made with mRNA complexed with indicated liposomes at different RNA/L charge ratios; (**B**), **top** – Impact of MDX combined with mRNA at mRNA/MDX weight ratio of 1/2 on indicated lipoplexes (LX) made at RNA/L charge ratio of 1/1.5; **bottom -** mRNA incubated with MDX at different mRNA/MDX weight ratios.

The formation of LXs was evaluated by preparing mRNA-liposome complexes at RNA/L charge ratios of 1/0.5, 1/1, 1/1.5 and 1/2. The mRNA encapsulation efficiency results using the Ribogreen Assay are seen in Table 2. The Ribogreen assay results are consistent with the agarose gel electrophoresis qualitative results (Fig. 1A), demonstrating that at RNA/L charge ratio of 1/0.5 all formulations have incomplete encapsulations (90.1, 94.8 and 90.6 for LX1, LX2 and LX3, respectively). Furthermore, the complexation of LX3 at RNA/L charge ratio of 1/1 was still incomplete. As seen in Fig. 1A, the intensity of the band in the agarose gel well depends on the strength of the interaction between mRNA and liposomes, being related to the level of Ribogreen exclusion. At different ratios tested, all liposomes were able to have a strong interaction with mRNA. mRNA complexed with lip 2 (LX2) exhibited the strongest interaction across all RNA/L charge ratios. At ratio 1/0.5, the intensity of the band was in the following order lip1 > lip3 > lip2, indicating a weaker interaction of mRNA with lip1 than with lip3, whereas the best interaction was obtained with lip2. Thus, to ensure complete mRNA complexation, in the next experiments, all LXs were uniformly prepared with the ratio 1/1.5.

**Table 2:** Encapsulation efficiency (%) of mRNA at different RNA/L charge ratios for lipoplex formulations LX1, LX2, and LX3.

Encapsulation efficiency %			
RNA/L charge ratio	LX1	LX2	LX3
1/0.5	90.1	94.8	90.6
1/1	91.1	100	94.0
1/1.5	94.8	100	97.8
1/2	94.2	98.9	99.4

### 3.2 MDX incorporation on lipoplexes

We evaluated whether the incorporation of MDX in LX could improve their muco-penetrating properties. To our knowledge, the preparation of such formulations has never been reported. First, we assessed the impact of the presence of MDX on the complexation between mRNA and liposomes. Our data show that both the complexation as well as the stability of the mRNA were unaffected (Fig. 1B, top). When mRNA was incubated with MDX at different ratios, no complexation of mRNA was observed as confirmed by the band migration of free mRNA (Fig. 1B, bottom). This is expected as MDX, being neutral [4], it cannot complex with mRNA.

**Fig. 2:**
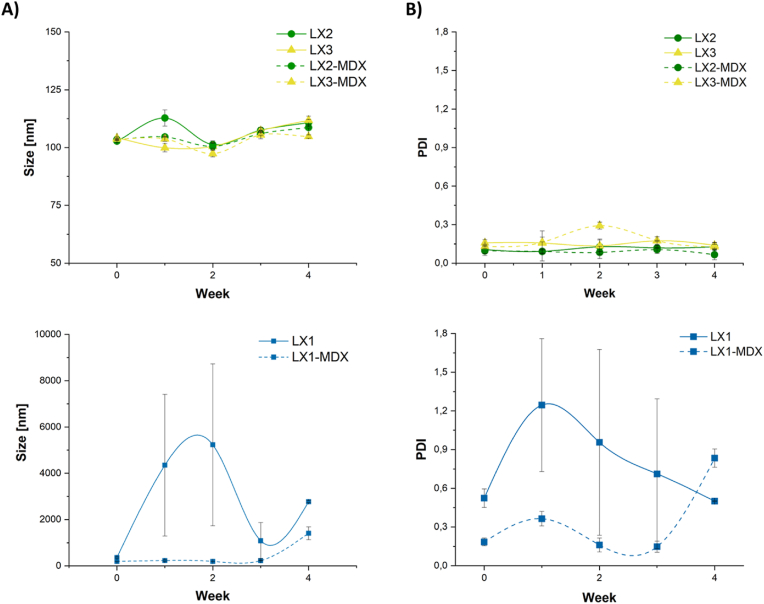
**Size stability**: LXs comprising 5 μg of mRNA at RNA/L charge ratio of 1/1.5 and LX-MDX: 5 μg of mRNA prepared at RNA/L charge ratio of 1/1.5 and RNA/MDX weight ratio of 1/2 were stored at 4 °C and their size was monitored by DLS at indicated times. DLS measurements are the mean of three experiments. (**A**), LX size; (**B**), LX polydispersity index (PDI) throughout one month of storage.

Concerning the size and aggregation throughout time, Fig. 2A (top) shows that for LX2 and LX3, the Z average diameter range is approximately 100 nm, regardless of the presence of MDX, remaining consistent over 4 weeks. Moreover, their PDI values are low and range from 0.092 to 0.128 for LX2 and 0.136 to 0.174 for LX3 (Fig. 2B, top). In contrast, the size of LX1 comprising only KLN25 and MM27 increased with time, up to 5000 nm after 2 weeks, indicating that LX1 was not stable and probably aggregated with time (Fig. 2A (bottom)). Interestingly, a stabilization effect of LX1 size was observed when MDX was introduced into the formulations. There is also a positive influence on their PDI values, which are also stabilized for 3 weeks (Fig. 2B, bottom). These data are in line with those reported by Gurtuk et al. [4] who included MDX in liposomal formulations by bonding it covalently to DSPE-PEG. They also found a stabilizing effect for a period of 5 months at 4 °C, as indicated by minimal changes in particle size and PDI [4]. Despite the difference in both types of formulations and the procedure by which MDX was included, the same behavior is observed for LX2 and LX3.

The zeta potential data provided in Table 3 illustrates the impact of liposome complexation and MDX coating on the surface charge of the LXs. The highest impact is observed for lip1 (LX1), with a decrease in its zeta potential from +38.34 mV (Lip1) to +4.20 mV (LX1), and further to +0.97 mV (LX1-MDX). For LX2 and LX3, there is no meaningful change in the charge (Lip2 from +34.02 mV to +19.6 mV and further decreasing to +17.52 mV and Lip3 from +23.92 mV to +10.44 mV and then increasing to +12.07 mV). This outcome could be due to the distinct composition of LX2 and LX3, in comparison to LX1. Containing imidazole lipids, DOPE and PEGylated lipids are well-documented for their roles in stabilizing particles and reducing the sensitivity of surface charge fluctuations [17,18]. These components likely mitigate significant changes in zeta potential upon the introduction of maltodextrin, which could explain the minimal impact of MDX on zeta potential observed in LX2 and LX3. In contrast, the absence of these stabilizing lipids in LX1 may increase its susceptibility to interaction with free imidazole-containing lipids, thus leading to surface charge alterations, resulting in a pronounced decrease in zeta potential. The reduction of the surface charge of LX1-MDX suggests that the presence of MDX in the formulation shields the cationic charge to a variable degree, according to the structure of the liposomes. The same phenomenon has been observed by Gurtuk et al. [4], where the incorporation of an MDX-modified lipid into a liposomal formulation led to a more neutral overall charge [4].

**Table 3:** ζ potential of liposomes, lipoplexes and MDX-Lipoplexes. LXs: 5 μg of mRNA prepared at RNA/L charge ratio of 1/1.5, LX-MDX: 5 μg of mRNA prepared at RNA/L charge ratio of 1/1.5 and RNA/MDX weight ratio of 1/2. Samples were diluted x250 in NaCl 10 mM.

Liposomes	ζ potential (mV)	Lipoplexes	ζ potential (mV)	Lipoplexes-Maltodextrin	ζ potential (mV)
***LIP1***	38.34 ± 0.83	***LX1***	4.20 ± 1.60	***LX1-MDX***	0.97 ± 2.20
***LIP2***	34.02 ± 0.12	***LX2***	19.60 ± 2.4	***LX2-MDX***	17.52 ± 0.20
***LIP3***	23.92 ± 1.25	***LX3***	10.44 ± 0.39	***LX3-MDX***	12.07 ± 0.92

The physicochemical features of formulations are particularly interesting knowing that the interaction of particles administered by the nasal route and their mucociliary clearance depend on surface properties and particle size. Particles larger than 500 nm are often immobilized, whereas 100–200 nm diameter particles penetrate the human respiratory mucus rapidly [19]. Moreover, positively charged delivery vehicles easily get trapped in anionic mucin glycoproteins, hindering their penetration through the mucus barrier and potentially affecting therapeutic efficacy. In contrast, neutral particles exhibit higher average transport rates [19]. Gurtuk et al. [4] also observed that their MDX-liposomes have improved the bioavailability and uptake compared to plain liposomes.

**Fig. 3:**
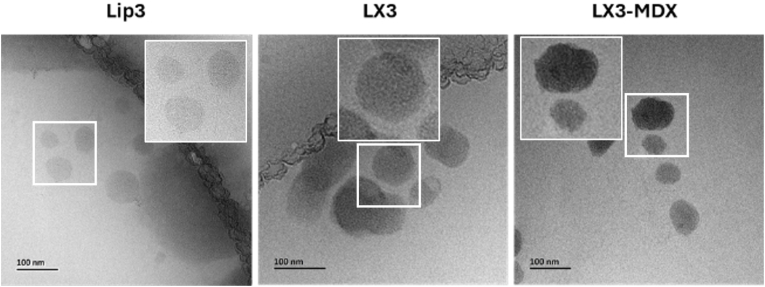
Cryogenic electron microscopy micrographs illustrating structural differences among lip3, LX3 and LX3-MDX at pH 7.4. LX comprised 5 μg of mRNA at RNA/L charge ratio of 1/1.5, LX-MDX comprised 5 μg of mRNA at RNA/L charge ratio of 1/1.5 and RNA/MDX weight ratio of 1/2. Scale bars represent 100 nm.

Cryo-EM analysis was conducted to assess the morphology of LX and LX-MDX (Fig. 3). Cryo-EM structural analysis at the studied pH reveals a thin rim of the lamellar cationic liposomes structure of lip3 , also seen in the work of Ngalle et al. [20] The mRNA complexation resulted in more dense layers, as observed for LX. LX-MDX has roughly the same structure, but with much denser layers due to MDX colocalization on the surface of the LX.

Confocal microscopy observation was also performed on LX made with FITC-MDX and Rho-labeled lip2. The merged images revealed yellow spots due to an overlapping of the red (Rho-lip2) and green (FITC-MDX) spots, confirming MDX and LX colocalization (Figure S2).

### 3.3 mRNA delivery in epithelial airway cells

To determine the efficiency of LX-MDX to deliver and express mRNA in airway epithelial cells, we used the NCI-H292 airway epithelial cell line as a cell model. These cells are able to form a cell monolayer mimicking the lung epithelium [3]. To first achieve an optimal mRNA expression and minimize potential toxicity, an initial screening was performed to determine the most suitable mRNA type and quantity. Therefore, two types of mRNA were considered, namely non-modified and modified mRNA (5 methoxy uridine) and three different quantities of mRNA (0.5 μg, 1 μg, and 2 μg) were used to prepare the formulations.

**Fig. 4:**
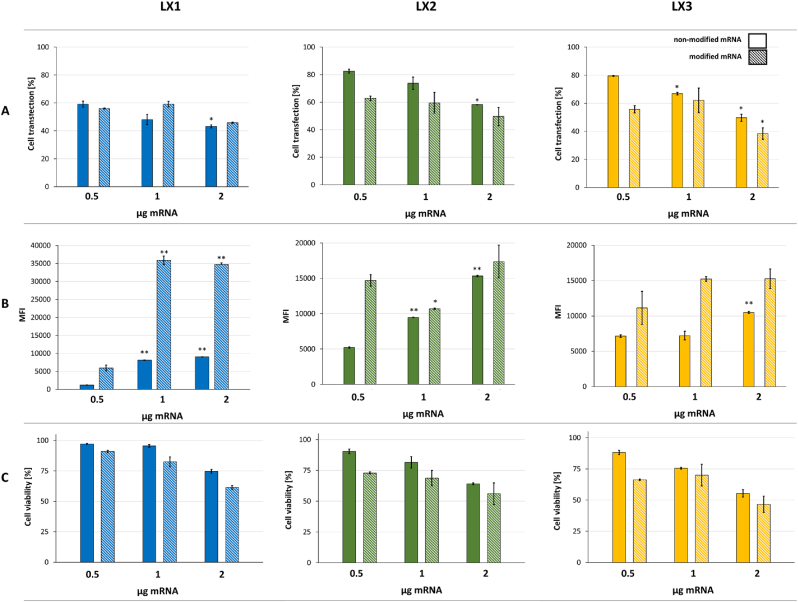
**mRNA transfection efficiency:** Airway epithelial cells were transfected with LXs (RNA/L charge ratio 1/1.5) comprising either 0.5, 1 or 2 μg of (full bars) non-modified or (striped bars) modified mRNA encoding eGFP. (**A**) percentage of eGFP positive cells. (**B**) mRNA translation: MFI corresponds to the means fluorescence intensity of eGFP and (**C**) cell viability was evaluated 24 h post-transfection. Data are presented as mean ± SD of experiments performed in triplicates (n = 3). ∗ <0.05. ∗∗p < 0.01 between all sample groups.

As shown in Fig. 4A, all formulations were able to efficiently deliver mRNA in NCI-H292 airway epithelial cells. The percentage of transfected cells with LX1, LX2 and LX3 decreased (from 60 % to 40 % for LX1, from 80 % to 50 % for LX2 and from 80 % to 40 % for LX3) when the amount of mRNA increased from 0.5 μg to 2 μg. In addition, the use of modified mRNA slightly decreased the percentage of transfected cells when using LX2 and LX3 comprising 0.5 and 1 μg of mRNA, while in the case of LX1, the opposite trend was observed. In this case, the mRNA translation efficiency increased (represented by MFI as mean fluorescence intensity) up to 4-fold with LX1, as shown in Fig. 4B. *In vitro* transcribed mRNA is known to induce the activation of innate immune system sensors leading to their degradation and/or blockade of the translation [1,[21], [22], [23]]. It is worth noting that this processing as well as the cell mortality and translation efficiency of mRNA are highly dependent on the structure of mRNA, the formulation used and the cell type.

Fig. 4C shows the cell viability assessed upon 24 h of transfection with the indicated lipoplexes. Cytotoxicity was dependent on the dose of mRNA. Notably, LX1 exhibited a lower cytotoxic effect on cells compared to formulations containing other liposomes (lip2 and lip3), which is consistent with the observed decrease in percentage of cell transfection. Surprisingly, our data revealed concentration-dependent cytotoxicity associated with modified mRNA LXs compared to those made with non-modified mRNA (for instance: compared to non-modified mRNA, formulations (LX1, LX2, and LX3) containing 0.5 μg of modified mRNA exhibited decreased cell viability by 7 %, 17 %, and 22 %, respectively).

Since reducing cytotoxicity is a key priority for intranasal delivery strategies, subsequent experiments concentrated on non-modified mRNA at a loading dose of 0.5 μg. This approach balanced a high transfection rate with acceptable MFI values within the tested range.

**Fig. 5:**
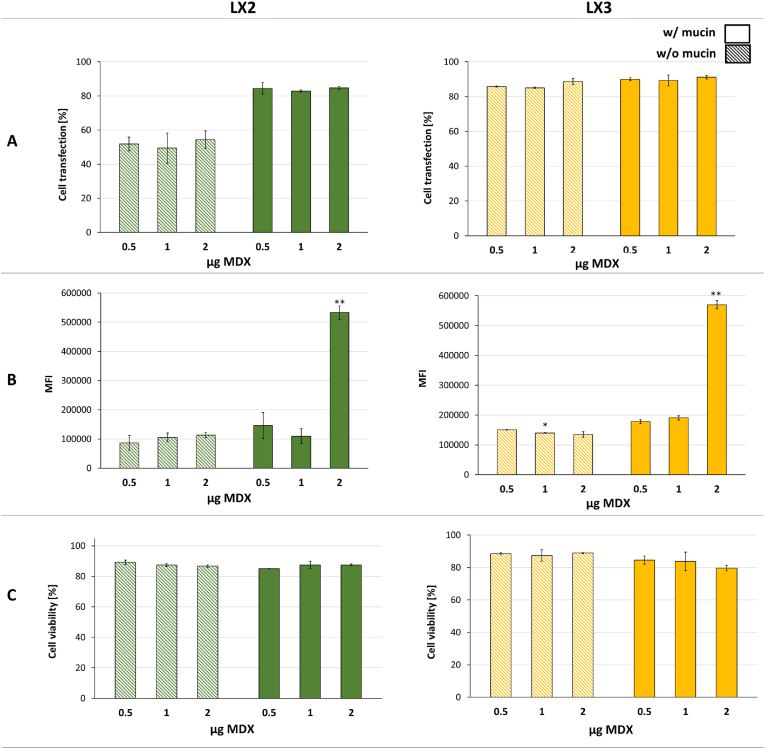
Impact of MDX dosage on the mucosal passage and mRNA transfection *in vitro*. Percentage of cell transfection (**A**), mean fluorescence intensity (**B**) and cell viability **(C)** were measured 24 h after treatment of airway epithelial cells with LXs comprising 0.5 μg of non-modified mRNA at an RNA/L charge ratio of 1/1.5 and RNA/MDX weight ratio of 1/0.5. 1/1 and 1/2. Experiments were divided into two groups, as indicated above the graphs. Striped bars represent samples not containing mucin. Data are presented as mean ± SD of experiments performed in triplicates (n = 3). ∗ <0.05. ∗∗p < 0.01 between all sample groups. w/o: without; w: with.

To assess the impact of several MDX dosages on transfection efficiency, experiments were conducted using LX2, LX2-MDX, LX3, and LX3-MDX formulations on mucin-covered airway epithelial cells (Fig. 5). Successful mRNA transfection was observed across all formulations, with a percentage of transfection ranging between 50 % and 90 %, as indicated in Fig. 5A. Notably, in the absence of mucin, LX2-MDX formulations achieved transfection rates of only 49 %–54 %, while LX3-MDX maintained high transfection efficiencies of around 85 %. Additionally, the MFI remained stable across the three dosages tested in the absence of mucin. For both LX2 and LX3, the presence of mucin did not significantly affect the percentage of transfected cells across different MDX dosages. However, coating LX2 and LX3 with 2 μg of MDX resulted in 4.8- and 2.9-fold higher protein expression in the presence of mucin as indicated by the eGFP expression (MFI) (Fig. 5B), respectively, compared to formulations with half the dosage (1 μg of MDX). In the case of 2 μg MDX, this outcome was attributed to an excess of resources available for cellular metabolism. When transfection was performed on mucin-covered epithelial monolayers, MDX did not significantly alter cell viability, with cell mortality reaching a maximum of 16 % for LX2-MDX (0.5 μg MDX) and 20 % for LX3-MDX (2 μg MDX) (Fig. 5C). These findings confirm that MDX is generally safe and biocompatible across all tested dosages, making it a viable option for gene delivery without inducing significant cytotoxicity.

**Fig. 6:**
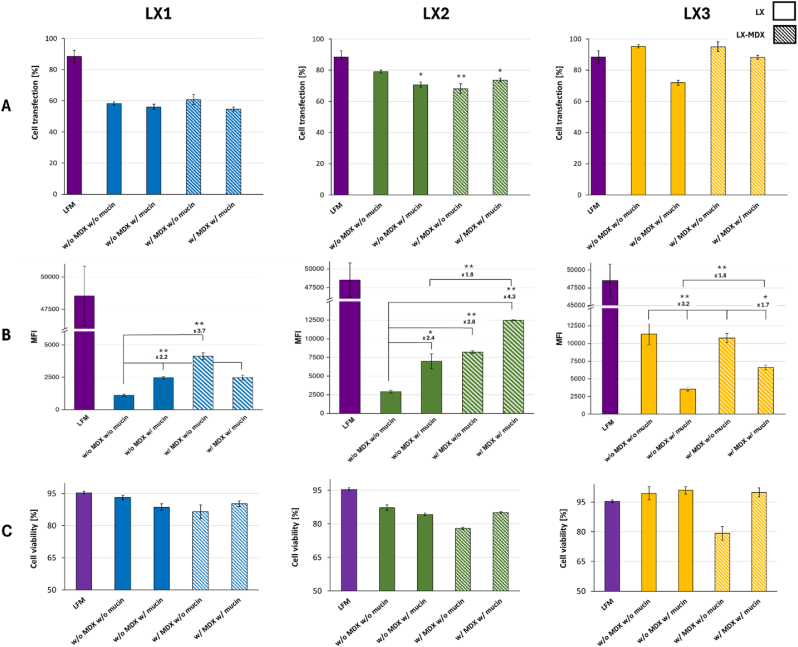
Impact of MDX on the mucosal passage and mRNA transfection *in vitro*. Percentage of cell transfection (**A**) and mean fluorescence intensity (**B**) were measured 24 h after treatment of airway epithelial cells with LXs comprising 0.5 μg of non-modified mRNA at an RNA/L charge ratio of 1/1.5 and RNA/MDX weight ratio of 1/2. Experiments were divided into four groups, as indicated under the graphs. Striped bars represent samples containing MDX. Data are presented as mean ± SD of experiments performed in triplicates (n = 3). ∗ <0.05. ∗∗p < 0.01 between all sample groups, excluding LFM. w/o: without; w: with.

To assess the impact of mucin and the benefit that could be gained with MDX presence on the transfection efficiency and gene expression. Experiments were conducted on airway epithelial cells covered with mucin (Fig. 6). A successful mRNA transfection was obtained in a range of 50–95 % of transfected cells with all LXs, thus leading to successful eGFP expression shown by the value of mean fluorescence intensity (Fig. 6B). It is worth noting that for LX1 no clear impact of the influence of MDX in the presence of mucin was observed for the percentage of transfected cells. Whilst a maximum of 2.2-fold higher translation was obtained when lipoplexes were coated with MDX. When the transfection was made on a monolayer of epithelial cells covered with mucin the presence of MDX in the formulation did not alter the cell viability (16.6 % of cell mortality at the highest for LX3-MDX) (Fig. 6C), however without it and without mucin, LX2 exhibited the most elevated toxicity of 13 %. Nonetheless, in the presence of mucin, the data confirms the fact that MDX is generally considered safe and biocompatible [24], making it a suitable option for gene delivery without causing significant toxicity or adverse effects on cells. The MDX coating led to a 2.8 % and 16.2 % increase in H292 cells transfection for LX2 and LX3, respectively. While the transfection efficiency (%) increased substantially for LX3 compared to LX2, the change in translation efficiency (MFI) of MDX-covered LX2 and LX3 uniformly increased 1.8-fold for both formulations. This suggests that the presence of MDX on the surface of LXs had an overall positive impact on the LXs, effectively carrying mRNA through the mucin layer, allowing LX endocytosis and successful gene expression within the cells (Fig. 6B). The results indicate that using MDX as a coating material for LXs can improve their efficiency in passing through the mucosal layer, delivering mRNA and promoting successful gene expression, making it a promising strategy for nasal gene delivery applications.

While the exact mechanism is not yet understood, it is hypothesized that the presence of maltodextrin on the surface of LXs renders them less hydrophobic and therefore facilitates the penetration of the mucus layer covering epithelial cells [25]. Confirming the mechanism could enhance the efficient delivery of mRNA to the underlying cells, leading to improved gene expression and therapeutic outcomes. Further research is required to fully elucidate the mechanistic role of maltodextrin in enhancing mRNA delivery through the mucus barrier.

### 3.4 Kinetics of mRNA delivery and translation

**Fig. 7:**
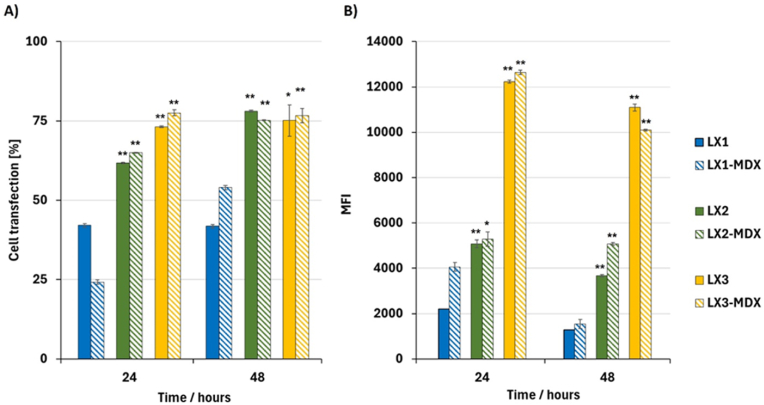
Impact of MDX on the intracellular trafficking of eGFP-mRNA throughout time. Percentage of positive eGFP cells (**A**) and encoded protein production (presented as MFI) (**B**) were measured at 24 and 48 h after transfection of airway epithelial cells with LXs comprising 0.5 μg of non-modified mRNA at an RNA/L charge ratio of 1/1.5 and RNA/MDX weight ratio of 1/2. Data are presented as mean ± SD of experiments performed in triplicates (n = 3). ∗ <0.05. ∗∗p < 0.01 between all sample groups.

To gain insights into the intracellular delivery of mRNA and the expression of eGFP over time, kinetic studies were conducted at 24 and 48 h. Epithelial cells were exposed to indicated formulations, coated or not with MDX (Fig. 7). Regarding the kinetics of the produced eGFP, the MFI values at 24 h clearly demonstrate the advantageous influence of MDX in the formulations. Specifically, LX1 exhibited a 2-fold increase in MFI (Fig. 7B). While the percentage of transfected cells in the case of LX3 and LX3-MDX remained stable at 24 h and 48 h (Fig. 7A), the MFI of LX3-MDX dropped lower than that of LX3 at 48 h. Interestingly, LX2-MDX sustained its MFI value even after 48 h, suggesting that this formulation experiences a slower endosomal escape and maintains a more sustained production of eGFP. These observations align with previous research on the beneficial effects of polysaccharides such as dextran in improving intracellular antigen delivery, endosomal escape and enhancing gene expression [26]. These findings emphasize the potential benefits of incorporating MDX on the lipoplexes for improving intracellular delivery kinetics and prolonging the expression of the delivered gene [26]. In addition, the incorporation of MDX in some formulations sustained the production of the encoded protein. Our results agree with those reported by Patel et al. [11]who investigated lipid formulations comprising *β*-sitosterol, DOPE, and cholesterol, demonstrating that the inclusion of all three compounds resulted in increased transfection percentages. Overall, our results provide valuable insights into the role of MDX in improving sustained mRNA delivery for some formulations and highlighting the potential importance of formulation components in enhancing transfection outcomes.

### 3.5 Uptake and intracellular trafficking of mRNA

mRNA requires a vector to facilitate efficient cellular uptake; therefore, the internalization and intracellular trafficking of mRNA depend on the type of delivery vehicle used. Cationic delivery vehicles typically interact with anionic proteoglycans and enter cells via endocytosis [27]. Furthermore, it was previously demonstrated that MDX-coated LXs were highly endocytosed via the Clathrin-dependent pathway, especially for nanoparticles with diameters less than 200 nm [27,28]. After internalization, the delivery vehicles are transported through early endosomes, which mature into late endosomes (pH 5–6) and eventually into lysosomes (pH ∼4.5) containing nucleases and degradative enzymes. Thus, mRNA must escape into the cytosol (pH ∼7.4) at an early stage to avoid lysosomal degradation [15,26].

**Fig. 8:**
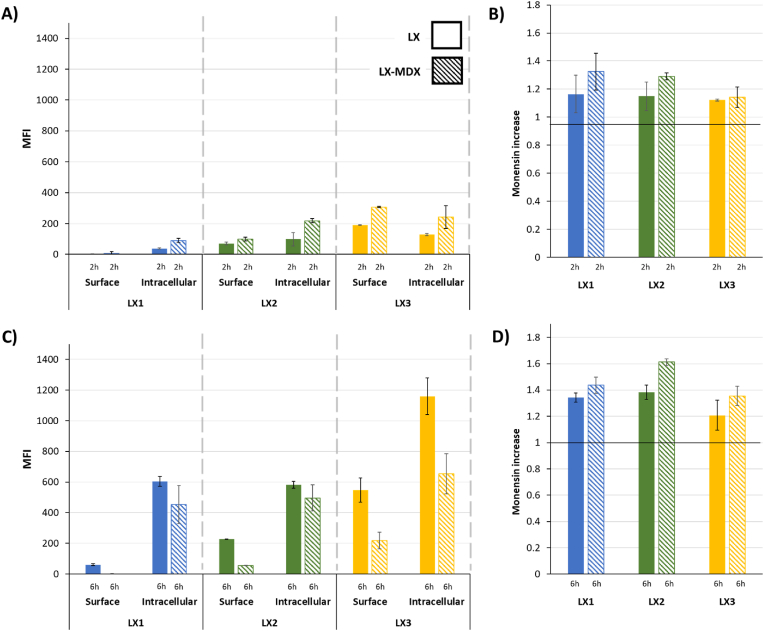
Impact of MDX on the intracellular trafficking of FITC-mRNA was measured at 2 and 6 h after transfection of airway epithelial cells with LXs comprising 0.5 μg of non-modified mRNA at an RNA/L charge ratio of 1/1.5 and RNA/MDX weight ratio of 1/2. Mean fluorescence intensity was followed on the surface and intracellularly (**A** and **C** at respectively 2 h and 6 h) and also in the endosomes (**B** and **D** at respectively 2 h and 6 h). Striped bars represent samples containing MDX. Data are presented as mean ± SD of experiments performed in triplicates (n = 3).

As seen in Fig. 8, the experimental assay was divided into three segments to examine and quantify the distribution of FITC-labeled mRNA on the cell surface, within the intracellular space, and trapped in endosomes. To achieve this, cellular association of LXs was assessed using a fluorescence quenching assay with trypan blue. Briefly, cells were incubated with LXs, followed by treatment with trypan blue to quench the fluorescence of cell surface-bound, fluorescein-labeled LXs. The difference in fluorescence intensity before and after trypan blue treatment represented the cell surface-associated LX fraction ("surface"). To quantify total cellular uptake and lysosomal localization of LXs, cells were incubated with LXs followed by treatment with monensin (0.0354 mg/mL in PBS, pH 7.4) for 30 min on ice. Monensin, an ionophore, permeabilizes cells and neutralizes acidic compartments, leading to increased fluorescence intensity of LXs previously residing in these acidic environments. Total cellular uptake was determined as the difference in mean fluorescence intensity measured after monensin treatment and fluorescence corresponding to surface binding. The presence of MDX had a beneficial effect on the lipoplex formulations during the time studied points.

The cellular uptake data (Fig. 8 and Figure S3) revealed an interplay between LX surface properties and internalization kinetics. Initially, all formulations containing MDX exhibited a higher LX presence on the cell surface compared to their non-coated counterparts (2.5-, 1.4-, and 2.6-fold increase, for LX1, LX2 and LX3 formulations, respectively). This suggests that MDX might influence initial adsorption to the cell surface. However, this trend reversed by the 6-h time point. Here, MDX-containing formulations displayed significantly lower fluorescence intensity on the cell surface (60-, 4.1-, and 2.5-fold decrease for LX1, LX2 and LX3 formulations respectively), indicating that MDX may promote LX internalization over time.

Interestingly, the intracellular LX content mirrored the surface trends. At the 2-h mark, MDX-coated LXs displayed a higher intracellular signal compared to non-coated LXs (2.4-, 2.2-, and 1.9-fold increase). This further supports the hypothesis that 10.13039/100010051MDX might enhance LX uptake. However, by the 6-h time point, the situation reversed, with MDX-coated samples exhibiting lower intracellular fluorescence (1.3-, 1.2-, and 1.7-fold decrease). These observations suggest that MDX may facilitate LX uptake, and it could also influence subsequent intracellular trafficking pathways. This is further supported by the monensin increase data (Fig. 8B and D), where all MDX-containing formulations displayed higher fluorescence intensity. This finding aligns with the hypothesis of MDX having an overall positive impact on the delivery and expression of mRNA, leading to superior levels of mRNA expression in the cells.

### 3.6 Lipoplexes cellular internalization kinetics

**Fig. 9:**
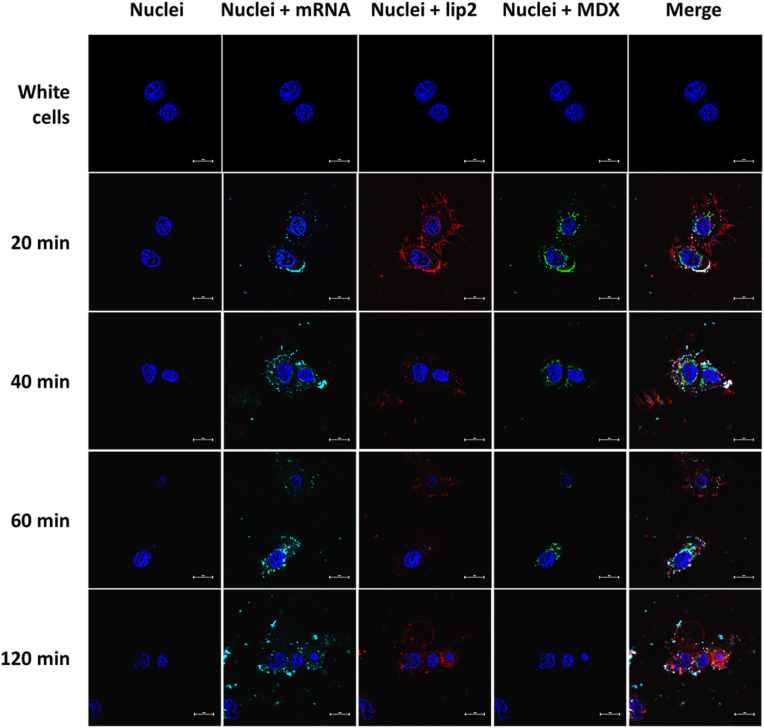
Fluorescent images of live airway epithelial cells captured at 20. 40. 60 and 120 min after LX treatment. LX comprising 0.5 μg of non-modified mRNA at an RNA/L charge ratio of 1/1.5 and RNA/MDX weight ratio of 1/2. Blue indicates nuclei, red: liposome, green: MDX and cyan: mRNA. Scale bar represents 10 μm. (For interpretation of the references to colour in this figure legend, the reader is referred to the Web version of this article.)

A detailed examination of the internalization kinetics and intracellular trafficking of mRNA and MDX utilizing confocal microscopy was additionally conducted. To achieve this, we prepared samples containing Cy5-labeled mRNA, FITC-labeled MDX, and rhodamine-labeled lip2. These samples were exposed to airway epithelial cells for a 2-h period. Subsequently, multiple images were captured to analyze the dynamics of the interactions within the cells. Fig. 9 reveals a difference in the intracellular behavior of MDX compared to the LX. In this study, the kinetics of MDX-coated LX can be followed by the exhibited fluorescent signals indicating a rapid cellular uptake and a distinctive trafficking route. The green signal of MDX can be seen surrounding the nucleus, likely in the endoplasmic reticulum, where it would not be metabolized; however, it could be accumulating in the cytoplasm near the nucleus [29]. Over time, the green signal conversely attenuates and almost completely disappears after 60 min. Moreover, the initially widespread bright colocalization signal of Cy5, Rho and FITC on the cell surface becomes more localized, due to endosome formation. These observations suggest that MDX enters the cells through a different mechanism and undergoes separate intracellular dynamics compared to LX. Therefore, it is suspected that MDX lacked the ability to stimulate faster endocytosis [28]. However, it appears to provide additional resources to the cell, potentially explaining the enhanced protein production observed in previous experiments. This suggests that MDX's distinct intracellular dynamics could be a contributing factor to its accelerated effect on mRNA expression. More investigations are required to fully determine how MDX may influence the intracellular dynamic and localization of lipoplexes.

### 3.7 Impact of MDX on intranasal delivery of LXs in mice

Intranasal delivery offers a convenient and effective route for administering therapeutics as well as mucosal vaccines, stimulating interest in the development of particulate carrier systems for this route. This approach is especially attractive for engaging mucosal surfaces, which serve as the primary entry points for many pathogens, playing a key role in initiating immune and physiological responses.

**Fig. 10:**
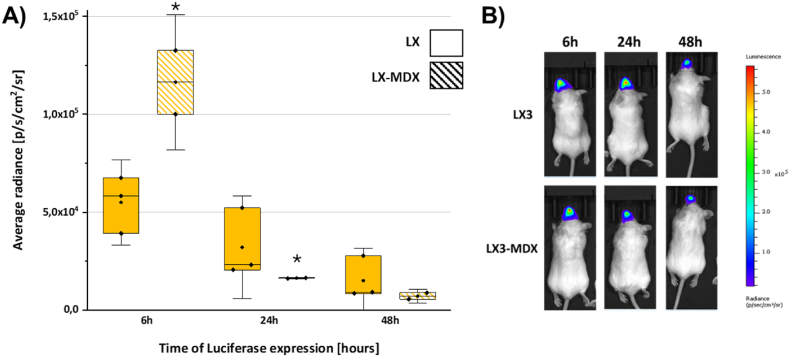
Impact of MDX on intranasal delivery of LXs. A) Luminescence expression was measured at 6. 24 and 48 h after intranasal injection on BALB/c mice with LX3 and LX3-MDX comprising 10 μg of F-Luc mRNA at an RNA/L charge ratio of 1/1.5 and RNA/MDX weight ratio of 1/2. B) Representative images of Firefly Luminescence in BALB/c Mice 6 h, 24 h and 48 h post intranasal injection. Striped bars represent samples containing MDX. ∗ <0.05. ∗∗p < 0.01 between all sample groups.

To evaluate the delivery and expression of mRNA *in vivo*, LXs formulated with MDX containing 10 μg of luciferase-encoding mRNA (F-Luc mRNA, 0.33 μg/μL) were administered to the nasal cavity of BALB/c female mice. The luciferase expression was tracked longitudinally *via* bioluminescence imaging using an IVIS imaging system. Following intranasal administration, bioluminescence was detectable at the inoculation site within 6 h (Fig. 10A and B), indicating successful transfection of mRNA-loaded particles. This signal persisted for up to 48 h, underscoring the sustained activity of the delivery system. Remarkably, the MDX coating significantly boosted F-Luc expression during the initial 6 h *in vivo* (Fig. 10B, bottom). Nevertheless, imaging at later time points revealed that F-Luc activity peaked at 24 h post-administration for LX3 (Fig. 10B, top), while MDX-coated LX3 exhibited slightly reduced F-Luc expression compared to its uncoated counterpart. By 48 h, bioluminescence declined, consistent with the anticipated short half-life of transfected mRNA *in vivo*. The data show that LX3-MDX leads to a higher initial protein expression at 6 h compared to LX3. This phenomenon correlates with the intracellular trafficking studies previously discussed, reinforcing the observation that MDX-modified LX effectively and rapidly deliver mRNA to the nasal cavity, thereby inducing faster early translation events. In some cases, such as with LX2-MDX at 48 h (Fig. 7B), a more sustained expression profile was observed. For LX3, the decay is less pronounced from 6 h to 24 h. However, the luminescence signals are quite similar between the two formulations at 24 h and 48 h.

Overall, these findings support the use of MDX-modified lipoplexes as a promising approach for mRNA delivery across mucosal barriers, enabling efficient localized protein expression. The results also suggest that MDX may enhance the potential of intranasal delivery strategies, laying the groundwork for more effective mucosal immunization approaches. However, exploring its role in vaccination lies beyond the scope of this study, as further optimization and evaluation are needed to fully assess the immunological impact of MDX as a mucosal adjuvant.

## 4 Conclusions

This study demonstrates the potential of maltodextrin (MDX)-coated lipoplexes for improving the delivery and expression of mRNA in airway epithelial cells. By enhancing cellular uptake and sustaining protein expression both *in vitro* and *in vivo*, MDX serves as an effective additive for mucosal gene therapy applications. MDX-coated lipoplexes showed increased transfection efficiency, improved particle stability, and prolonged protein expression, particularly in the presence of mucin, suggesting their suitability for overcoming barriers associated with mucosal surfaces. Moreover, MDX influences LX adsorption to the cell surface initially, promoting internalization over time. While the precise mechanism of action remains to be studied, it is hypothesized that MDX may render LXs less hydrophobic, which may facilitate mucus penetration and promote intracellular delivery. Moreover, the mucosal environment is dynamic, with pH and enzyme gradients. Non-covalent interactions allow maltodextrin to interact adaptively with mucins, hydrophilic layers, or surface proteins, improving mucosal penetration and diffusion.

The reported findings not only validate the potential of MDX-coated LXs for nasal gene delivery applications but also pave the way for further investigations into the underlying mechanisms driving this unique performance. We acknowledge that data gained from this study must not be generalized for all types of formulations and cell models.

## CRediT authorship contribution statement

**Bojan Kopilovic:** Writing – original draft, Methodology, Investigation, Formal analysis, Data curation. **Nabila Laroui:** Writing – original draft, Visualization, Methodology, Investigation, Formal analysis, Data curation. **Mathieu Berchel:** Writing – review & editing, Resources, Data curation. **Paul-Alain Jaffrès:** Writing – review & editing, Validation, Resources. **Patrick Midoux:** Writing – review & editing, Supervision, Methodology, Formal analysis, Conceptualization. **Mara G. Freire:** Writing – review & editing, Validation, Supervision, Funding acquisition, Formal analysis, Conceptualization. **Chantal Pichon:** Writing – review & editing, Validation, Supervision, Resources, Project administration, Funding acquisition, Conceptualization.

## Statement of significance

This study offers a novel method for improving messenger ribonucleic acid (mRNA) nasal administration by using histidylated liposomes modified with maltodextrin (MDX). Histidylated liposomes, known for their endosomal escape properties and efficient intracellular delivery, were optimized through MDX incorporation to overcome mucosal barrier. These MDX-coated lipoplexes exhibit improved mRNA stability, enhanced penetration through mucus, efficient cellular uptake, and sustained protein expression in airway epithelial cells. The combination of biocompatible materials and optimized surface properties makes this formulation a strong candidate for gene therapy applications targeting respiratory mucosa. The findings contribute to the development of non-invasive delivery platforms for localized protein expression.

## Declaration of competing interest

The authors declare that they have no known competing financial interests or personal relationships that could have appeared to influence the work reported in this paper.

## Data Availability

Data will be made available on request.
