# Supplementary material for: Maltodextrin-modified lipoplexes for enhanced mucosal penetration and efficient mRNA delivery
Source: Mater Today Bio. 2025 Jun 13;33:101975. doi: 10.1016/j.mtbio.2025.101975 (PMC12221605; doi:10.1016/j.mtbio.2025.101975)
Supplement: Multimedia component 1 [file mmc1.docx]

Supporting Information

Maltodextrin-Modified Lipoplexes for enhanced Mucosal Penetration and Efficient mRNA Delivery

Bojan Kopilovic^1†^, Nabila Laroui^2,3†^, Mathieu Berchel^5^, Paul-Alain Jaffrès^5^, Patrick Midoux^2,3,^ Mara G. Freire^1^, Chantal Pichon^2,3,4*^

^1^ CICECO - Aveiro Institute of Materials, Department of Chemistry, University of Aveiro, 3810-193 Aveiro, Portugal

^2^ Centre de Biophysique Moléculaire (CBM), UPR 4301 CNRS, F-45071 Orléans, France

^3^ Inserm UMS 55 ART ARNm and University of Orléans, F-45100 Orléans, France

^4^ Institut Universitaire de France, 1 rue Descartes, F-75035 Paris, France

^5^ Univ Brest, CEMCA, UMR CNRS - UBO 6521, F-29200, Brest, France

[chantal.pichon@inserm.fr](mailto:chantal.pichon@inserm.fr)

† B. K. and N. L. have made equal contributions to this work.

Materials and methods

eGFP RNA sequence:

GGGAGACAAGCUUGCAUGCCUGCAGGUCGACUCUAGAGGAUCCACCGGUCGCCACCAUGGUGAGCAAGGGCGAGGAGCUGUUCACCGGGGUGGUGCCCAUCCUGGUCGAGCUGGACGGCGACGUAAACGGCCACAAGUUCAGCGUGUCCGGCGAGGGCGAGGGCGAUGCCACCUACGGCAAGCUGACCCUGAAGUUCAUCUGCACCACCGGCAAGCUGCCCGUGCCCUGGCCCACCCUCGUGACCACCCUGACCUACGGCGUGCAGUGCUUCAGCCGCUACCCCGACCACAUGAAGCAGCACGACUUCUUCAAGUCCGCCAUGCCCGAAGGCUACGUCCAGGAGCGCACCAUCUUCUUCAAGGACGACGGCAACUACAAGACCCGCGCCGAGGUGAAGUUCGAGGGCGACACCCUGGUGAACCGCAUCGAGCUGAAGGGCAUCGACUUCAAGGAGGACGGCAACAUCCUGGGGCACAAGCUGGAGUACAACUACAACAGCCACAACGUCUAUAUCAUGGCCGACAAGCAGAAGAACGGCAUCAAGGUGAACUUCAAGAUCCGCCACAACAUCGAGGACGGCAGCGUGCAGCUCGCCGACCACUACCAGCAGAACACCCCCAUCGGCGACGGCCCCGUGCUGCUGCCCGACAACCACUACCUGAGCACCCAGUCCGCCCUGAGCAAAGACCCCAACGAGAAGCGCGAUCACAUGGUCCUGCUGGAGUUCGUGACCGCCGCCGGGAUCACUCUCGGCAUGGACGAGCUGUACAAGUAAAGCGGCCGCGGAUCCCCGGGUACCGAGCUCGAAUUCUUAAUUAAAAAAAAAAAAAAAAAAAAAAAAAAAAAAAAAAAAAAAAAAAAAAAAAAAAAAAAAAAAAAAAAAAAAAAAAAAAAAAAAAAAAAAAAAAAAAAAAAAAAAAAAAAAAAAAAAAAAAAAAAAAAAAAAAAAAAAAAAAAAAAAAAAAAAAAAAAAAAAAAAAAAAAAAAAAA

Modification: 5MoU

Capping: ARCA and cap1

F Luc RNA sequence:

5’-AUGGAGGACGCCAAGAACAUCAAGAAGGGCCCCGCCCCCUUCUACCCCCUGGAGGACGGCACCGCCGGCGAGCAGCUGCACAAGGCCAUGAAGCGGUACGCCCUGGUGCCCGGCACCAUCGCCUUCACCGACGCCCACAUCGAGGUGGACAUCACCUACGCCGAGUACUUCGAGAUGAGCGUGCGGCUGGCCGAGGCCAUGAAGCGGUACGGCCUGAACACCAACCACCGGAUCGUGGUGUGCAGCGAGAACAGCCUGCAGUUCUUCAUGCCCGUGCUGGGCGCCCUGUUCAUCGGCGUGGCCGUGGCCCCCGCCAACGACAUCUACAACGAGCGGGAGCUGCUGAACAGCAUGGGCAUCAGCCAGCCCACCGUGGUGUUCGUGAGCAAGAAGGGCCUGCAGAAGAUCCUGAACGUGCAGAAGAAGCUGCCCAUCAUCCAGAAGAUCAUCAUCAUGGACAGCAAGACCGACUACCAGGGCUUCCAGAGCAUGUACACCUUCGUGACCAGCCACCUGCCCCCCGGCUUCAACGAGUACGACUUCGUGCCCGAGAGCUUCGACCGGGACAAGACCAUCGCCCUGAUCAUGAACAGCAGCGGCAGCACCGGCCUGCCCAAGGGCGUGGCCCUGCCCCACCGGACCGCCUGCGUGCGGUUCAGCCACGCCCGGGACCCCAUCUUCGGCAACCAGAUCAUCCCCGACACCGCCAUCCUGAGCGUGGUGCCCUUCCACCACGGCUUCGGCAUGUUCACCACCCUGGGCUACCUGAUCUGCGGCUUCCGGGUGGUGCUGAUGUACCGGUUCGAGGAGGAGCUGUUCCUGCGGAGCCUGCAGGACUACAAGAUCCAGAGCGCCCUGCUGGUGCCCACCCUGUUCAGCUUCUUCGCCAAGAGCACCCUGAUCGACAAGUACGACCUGAGCAACCUGCACGAGAUCGCCAGCGGCGGCGCCCCCCUGAGCAAGGAGGUGGGCGAGGCCGUGGCCAAGCGGUUCCACCUGCCCGGCAUCCGGCAGGGCUACGGCCUGACCGAGACCACCAGCGCCAUCCUGAUCACCCCCGAGGGCGACGACAAGCCCGGCGCCGUGGGCAAGGUGGUGCCCUUCUUCGAGGCCAAGGUGGUGGACCUGGACACCGGCAAGACCCUGGGCGUGAACCAGCGGGGCGAGCUGUGCGUGCGGGGCCCCAUGAUCAUGAGCGGCUACGUGAACAACCCCGAGGCCACCAACGCCCUGAUCGACAAGGACGGCUGGCUGCACAGCGGCGACAUCGCCUACUGGGACGAGGACGAGCACUUCUUCAUCGUGGACCGGCUGAAGAGCCUGAUCAAGUACAAGGGCUACCAGGUGGCCCCCGCCGAGCUGGAGAGCAUCCUGCUGCAGCACCCCAACAUCUUCGACGCCGGCGUGGCCGGCCUGCCCGACGACGACGCCGGCGAGCUGCCCGCCGCCGUGGUGGUGCUGGAGCACGGCAAGACCAUGACCGAGAAGGAGAUCGUGGACUACGUGGCCAGCCAGGUGACCACCGCCAAGAAGCUGCGGGGCGGCGUGGUGUUCGUGGACGAGGUGCCCAAGGGCCUGACCGGCAAGCUGGACGCCCGGAAGAUCCGGGAGAUCCUGAUCAAGGCCAAGAAGGGCGGCAAGAUCGCCGUGUGA- 3’

Modification: 5MoU

Capping: cap1

**Figure S1.** Chemical structure of the lipids used in this study.

**Results**


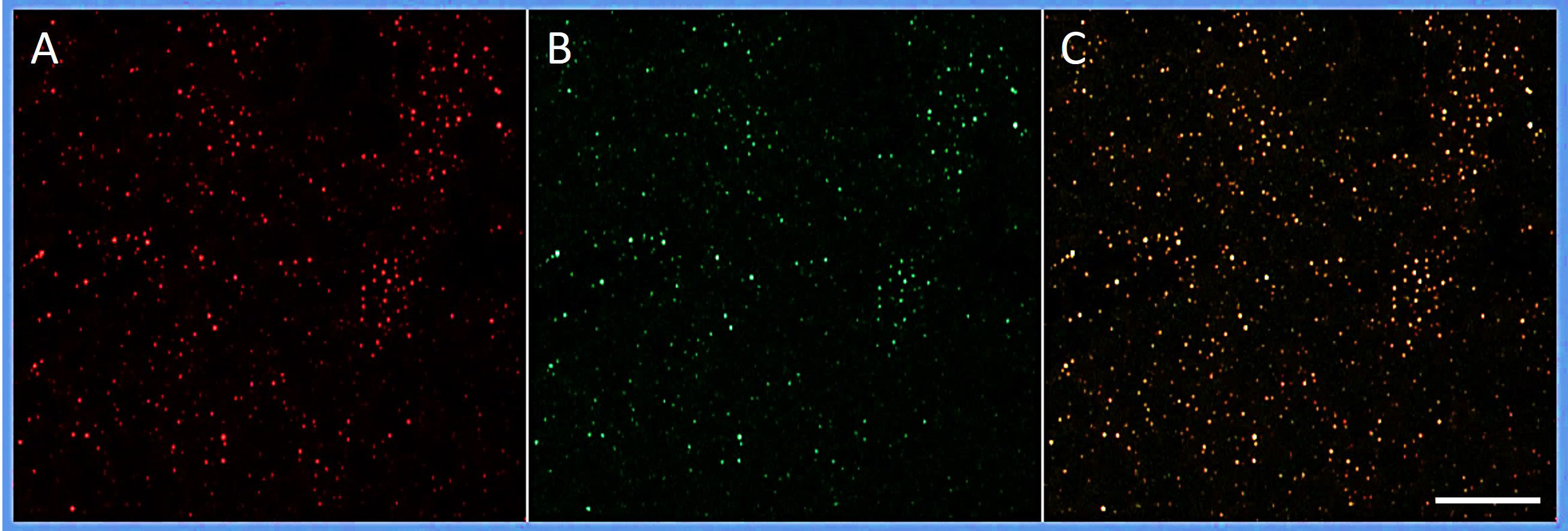


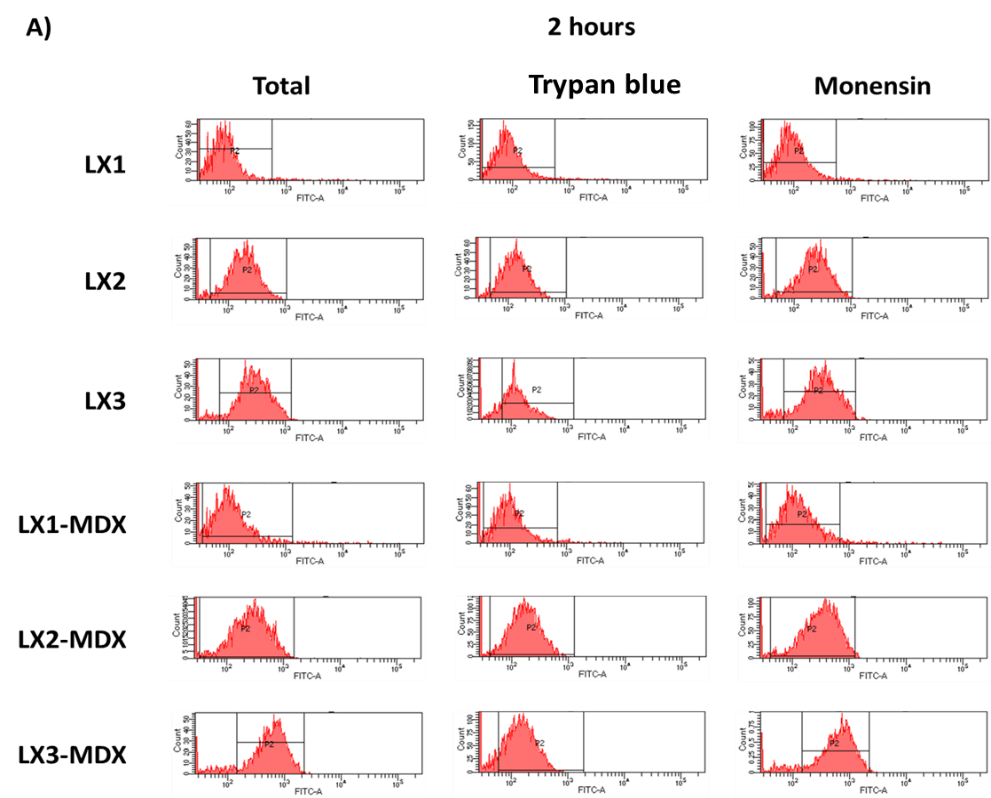
**Figure S2**. MDX and LXs colocalization assay using confocal microscopy. LXs comprising 0.5 µg of non-modified mRNA at an RNA/L charge ratio of 1/1.5, and RNA/MDX weight ratio of 1/2. A – Rhodamine-labelled lip2; B – FITC-MDX; C – merged representation. Scale bar represents 5 μm.

**
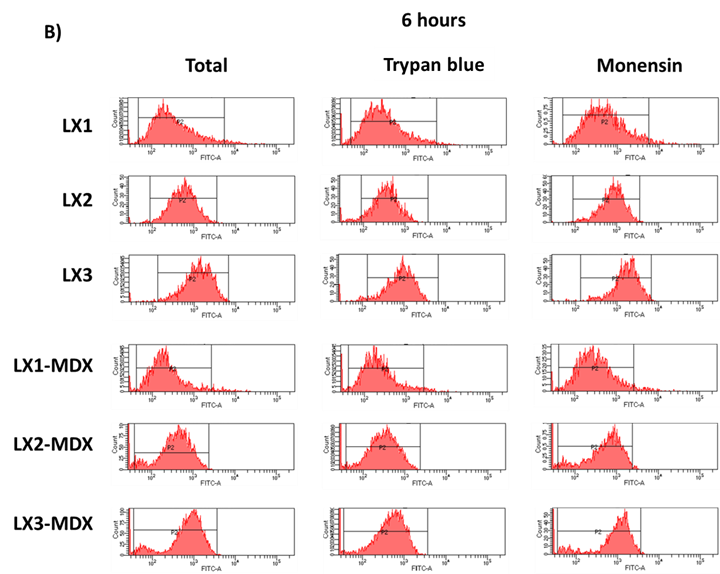
**

**Figure S3**. Histograms of H292 cells following the uptake of FITC-mRNA complexed to different formulations. Impact of MDX on the uptake measured at (**A**) 2 and (**B**) 6h after transfection of airway epithelial cells with LXs comprising 0.5 µg of non-modified mRNA at an RNA/L charge ratio of 1/1.5 and RNA/MDX weight ratio of 1/2.
